# Atractylenolide III Attenuates TGF-β1-Induced Myofibroblast Differentiation in Primary Human Corneal Keratocytes

**DOI:** 10.3390/molecules31214040

**Published:** 2026-11-09

**Authors:** Yura Choi, Byung Soo Koo, Choul Yong Park

**Affiliations:** 1 Department of Ophthalmology, Samsung Medical Center, Sungkyunkwan University School of Medicine, Seoul 06351, Republic of Korea; 2 Department of Oriental Neuropsychiatry, Graduate School of Dongguk University, Seoul 04620, Republic of Korea

**Keywords:** Atractylenolide III, corneal keratocyte, corneal fibrosis, TGF-β1, myofibroblast differentiation, α-SMA, SMAD2/3

## Abstract

Corneal stromal fibrosis is characterized by the transition of quiescent keratocytes into α-smooth muscle actin (α-SMA)-positive myofibroblasts and excessive extracellular matrix deposition, processes largely driven by transforming growth factor-beta 1 (TGF-β1) signaling. Atractylenolide III (ATL-III), a naturally derived sesquiterpene lactone, has demonstrated antifibrotic activity in several experimental models; however, its effects on human corneal keratocytes remain unclear. In this study, we investigated the effects of ATL-III on TGF-β1-induced myofibroblast differentiation in primary human corneal keratocytes. Cells were treated with TGF-β1 (10 ng/mL) alone or in combination with ATL-III (50 or 100 µM). Cell viability, fibrosis-associated gene expression, α-SMA expression and cytoskeletal organization, and intracellular signaling were evaluated using a cell viability assay, quantitative real-time PCR, immunofluorescence staining, and Western blot analysis. ATL-III did not significantly affect cell viability at concentrations up to 120 µM after 24 h, and cell viability remained unchanged under the 48 h treatment conditions used for the phenotypic analyses. TGF-β1 markedly increased the expression of ACTA2, COL1A1, CTGF, and Fibronectin (FN1). ATL-III significantly reduced ACTA2 expression at both 50 and 100 µM and reduced COL1A1 expression at 100 µM, whereas CTGF and FN1 showed decreasing trends without statistical significance. Immunofluorescence analysis showed attenuation of TGF-β1-induced α-SMA-associated filamentous organization following ATL-III treatment. Western blot analysis further demonstrated a significant reduction in α-SMA protein expression at 100 µM ATL-III. At the signaling level, ATL-III significantly reduced TGF-β1-induced SMAD2/3 phosphorylation at 50 µM, whereas AMPK, ERK1/2, and NF-κB phosphorylation were not significantly altered by ATL-III. Notably, p38 MAPK phosphorylation was further increased at 100 µM ATL-III despite attenuation of selected myofibroblast-associated features. These findings provide preliminary evidence that ATL-III attenuates selected features of TGF-β1-induced myofibroblast differentiation in primary human corneal keratocytes. The reduction in SMAD2/3 phosphorylation observed at 50 µM was associated with, but does not establish a causal relationship with, the phenotypic changes. In addition, p38 MAPK phosphorylation was increased at 100 µM, indicating that the signaling response was not uniformly concentration-dependent. Further protein-level, pathway-specific, and in vivo studies are required to determine the potential relevance of ATL-III to pathological corneal stromal remodeling.

## 1. Introduction

Corneal transparency is essential for vision and is maintained by the highly organized extracellular matrix (ECM) and the quiescent phenotype of corneal keratocytes [1,2]. Following corneal injury, quiescent keratocytes undergo phenotypic activation into fibroblasts and α-smooth muscle actin (α-SMA)-positive myofibroblasts, which are necessary for tissue repair [3,4,5]. However, persistent activation leads to excessive extracellular matrix deposition, resulting in stromal scarring and loss of corneal transparency, ultimately compromising visual function [4,5].

Transforming growth factor-beta (TGF-β) is a major regulator of corneal stromal wound healing and fibroblast-to-myofibroblast differentiation [5,6]. Sustained TGF-β signaling promotes α-SMA-positive myofibroblast differentiation and extracellular matrix remodeling through canonical SMAD signaling and interactions with non-canonical pathways, including MAPK signaling [6,7,8].Recent studies using primary human corneal stromal cells further support the central role of TGF-β signaling in the transition toward a myofibroblast phenotype [7,8].

In the clinical setting, current therapeutic approaches for corneal fibrosis mainly rely on corticosteroids and antifibrotic agents [6,9]. Although these treatments can reduce inflammation and fibrotic progression, their long-term use is limited by adverse effects including the risk of increased intraocular pressure, delayed corneal epithelial wound healing, and increased susceptibility to infection [9,10,11]. Accordingly, there is growing interest in identifying alternative therapeutic agents capable of suppressing pathological fibrosis while preserving physiological wound healing response [6,9].

Atractylodes macrocephala is a medicinal plant of the Asteraceae family and contains multiple classes of bioactive constituents, including sesquiterpene lactones and polysaccharides [12,13,14]. Atractylenolides I–III are chemically defined small-molecule sesquiterpene lactones that have been investigated for anti-inflammatory and organ-protective activities through multiple intracellular signaling pathways [12,13]. In contrast, Atractylodes polysaccharides comprise chemically heterogeneous macromolecular fractions whose biological properties vary according to extraction methods, molecular weight, monosaccharide composition, and structural characteristics [14]. ATL-III was selected for the present study not because it has been established as superior to other Atractylodes-derived preparations, but because it is a chemically defined compound with previously reported biological activity in signaling pathways relevant to cellular remodeling [13].

Previous studies have shown that ATL-III modulates cellular responses in several experimental disease models, including pulmonary, hepatic, cardiac, and intestinal remodeling [15,16,17,18]. ATL-III has also been reported to influence AMPK and other intracellular signaling pathways [18,19]. These findings provided the rationale for evaluating ATL-III in a corneal stromal cell model.

However, the effect of ATL-III on TGF-β1-induced myofibroblast differentiation in human corneal stromal cells has not been defined. Therefore, this study aimed to evaluate whether ATL-III alters TGF-β1-induced myofibroblast-associated phenotypic changes and to characterize signaling responses associated with ATL-III treatment.

## 2. Results

### 2.1. Effect of ATL-III on Cell Viability in Cultured Primary Human Corneal Keratocytes

**Figure 1:**
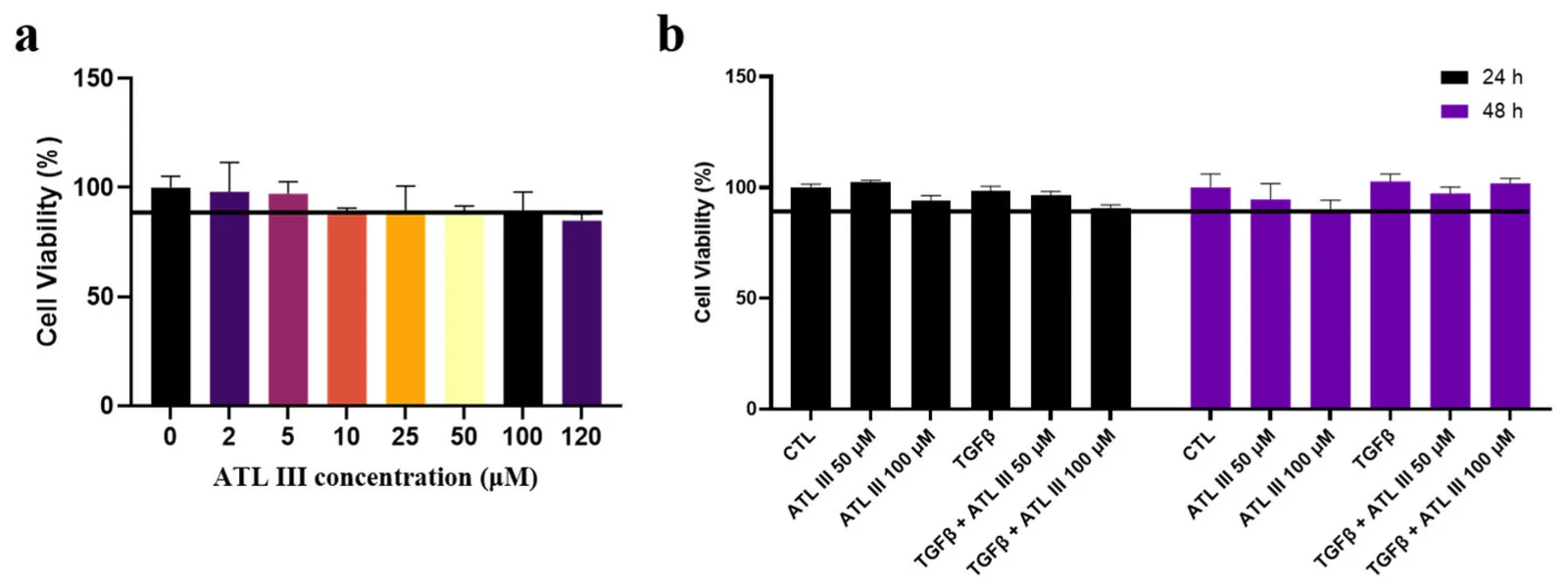
**Effect of ATL-III on cell viability in primary human corneal keratocytes.** (**a**) Cell viability after treatment with increasing concentrations of ATL-III (0, 2, 5, 10, 25, 50, 100, and 120 µM) for 24 h. (**b**) Cell viability after 24 h and 48 h of treatment with vehicle control, ATL-III (50 or 100 µM), TGF-β1 (10 ng/mL), and TGF-β1 in combination with ATL-III (50 or 100 µM). Cell viability is expressed relative to the untreated control group. *n* = 3 independent experiments. Data are presented as mean ± SEM. No statistically significant differences were observed among the experimental groups at either time point.

To determine the concentration range of ATL-III suitable for subsequent experiments, cell viability was first evaluated after 24 h of treatment with increasing concentrations of ATL-III (0, 2, 5, 10, 25, 50, 100, and 120 µM). No significant differences in cell viability were observed among the tested concentrations compared with the untreated control (Figure 1a). Based on the absence of detectable cytotoxicity across the tested concentration range, 50 and 100 µM ATL-III were selected as representative concentrations for subsequent phenotypic experiments. This initial viability screen was designed to assess cellular tolerance and was not intended to determine the minimum effective concentration of ATL-III. To further assess whether prolonged ATL-III exposure affected cell viability under the conditions used for the phenotypic analyses, cell viability was evaluated after 24 h and 48 h of treatment. No significant differences in cell viability were observed among the experimental groups at either time point (Figure 1b). These findings indicate that no detectable reduction in cell viability occurred under the ATL-III concentrations and exposure periods examined in this in vitro model.

### 2.2. ATL-III Attenuates TGF-β1-Induced Expression of Fibrosis-Associated Genes

**Figure 2:**
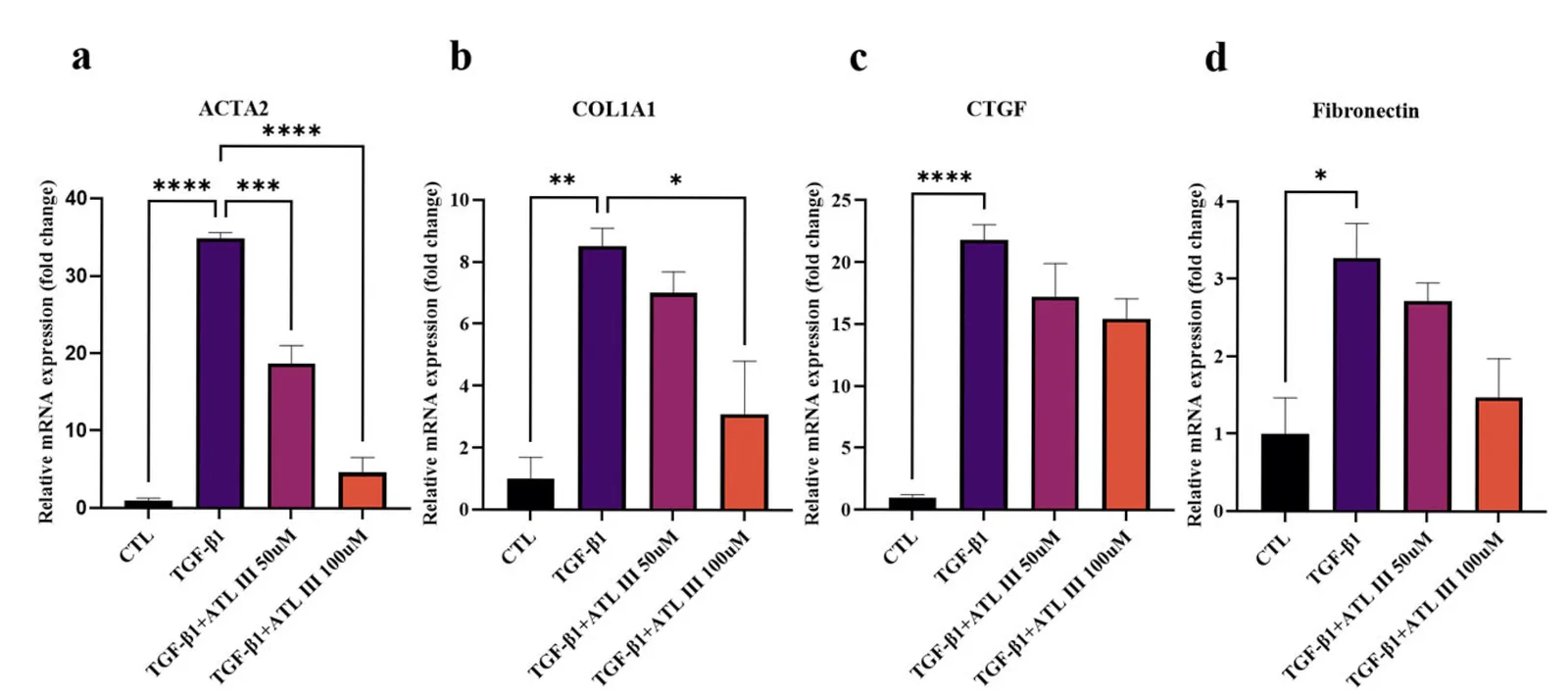
**Effects of ATL-III on TGF-β1-induced expression of fibrosis-associated genes in primary human corneal keratocytes**. Primary human corneal keratocytes were treated with TGF-β1 (10 ng/mL) alone or in combination with ATL-III (50 or 100 µM) for 48 h. Relative mRNA expression levels of (**a**) ACTA2, (**b**) COL1A1, (**c**) CTGF, and (**d**) Fibronectin (FN1) were determined by quantitative real-time PCR and normalized to GAPDH. n = 3 independent experiments. Data are presented as mean ± SEM. Statistical significance was determined by one-way ANOVA followed by Tukey’s multiple-comparison test. * *p* < 0.05, ** *p* < 0.01, *** *p* < 0.001, and **** *p* < 0.0001.

TGF-β1 treatment markedly increased the gene expression of α-SMA, COL1A1, CTGF, and FN1 compared with the untreated control (Figure 2). ATL-III treatment for 48 h significantly reduced TGF-β1-induced α-SMA expression at both 50 and 100 µM, with a greater reduction observed at 100 µM. COL1A1 expression was also reduced following ATL-III treatment, reaching statistical significance at 100 µM compared with TGF-β1 treatment alone. CTGF expression showed a decreasing trend following ATL-III treatment; however, the reductions at 50 and 100 µM were not statistically significant compared with TGF-β1 treatment alone. FN1 expression also showed a decreasing trend following ATL-III treatment, with a greater reduction observed at 100 µM; however, the differences compared with TGF-β1 treatment alone did not reach statistical significance. Collectively, these results indicate that ATL-III alters selected transcriptional features associated with TGF-β1-induced myofibroblast differentiation, with significant effects on ACTA2 and COL1A1 and nonsignificant decreasing trends in CTGF and FN1.

### 2.3. ATL-III Attenuates TGF-β1-Induced Myofibroblast Differentiation

**Figure 3:**
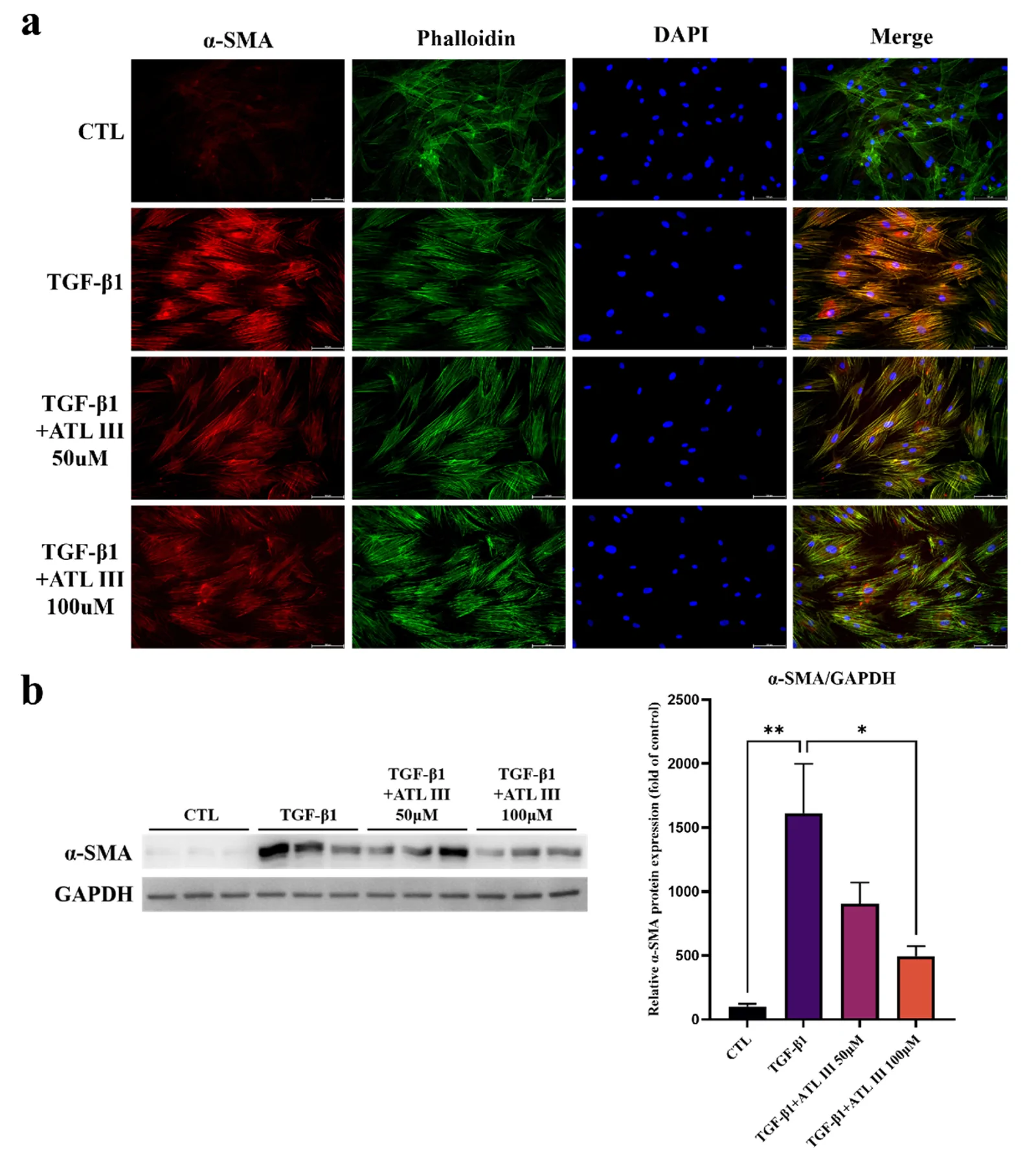
**ATL-III attenuates TGF-β1-induced myofibroblast differentiation in primary human corneal keratocytes**. Primary human corneal keratocytes were treated with TGF-β1 (10 ng/mL) alone or in combination with ATL-III (50 or 100 µM) for 48 h. (**a**) Representative immunofluorescence images of α-SMA (red), F-actin stained with phalloidin (green), and nuclei counterstained with DAPI (blue), with merged images shown in the right column. Experimental groups are arranged from top to bottom as untreated control (CTL), TGF-β1, TGF-β1 + ATL-III 50 µM, and TGF-β1 + ATL-III 100 µM. Images were acquired using identical acquisition settings across all experimental groups. Scale bar = 100 µm. (**b**) Representative Western blot images of α-SMA and GAPDH together with densitometric analysis of α-SMA protein expression normalized to GAPDH and expressed relative to the untreated control group. n = 3 independent experiments. Data are presented as mean ± SEM. Statistical significance was determined by one-way ANOVA followed by Tukey’s multiple-comparison test. * *p* < 0.05 and ** *p* < 0.01.

To further examine the effect of ATL-III on TGF-β1-induced myofibroblast differentiation, α-SMA expression and cytoskeletal organization were evaluated by immunofluorescence staining and Western blot analysis after 48 h of treatment (Figure 3).

Immunofluorescence staining showed relatively weak α-SMA immunoreactivity in control keratocytes, whereas TGF-β1 treatment markedly enhanced α-SMA staining and induced prominent filamentous α-SMA organization along the cellular cytoskeleton. ATL-III treatment attenuated the TGF-β1-induced α-SMA staining pattern, with less prominent α-SMA-associated filamentous organization observed in cells treated with 50 or 100 µM ATL-III compared with cells treated with TGF-β1 alone (Figure 3a). F-actin organization was visualized using phalloidin staining, and nuclei were counterstained with DAPI.

Western blot analysis further showed that TGF-β1 markedly increased α-SMA protein expression compared with the untreated control. ATL-III treatment reduced TGF-β1-induced α-SMA protein expression at both concentrations, with a greater reduction observed at 100 µM (Figure 3b). Densitometric analysis of α-SMA expression normalized to GAPDH confirmed a significant increase following TGF-β1 treatment. ATL-III treatment at 100 µM significantly reduced α-SMA expression compared with TGF-β1 treatment alone, whereas the reduction observed at 50 µM did not reach statistical significance (Figure 3b).

Together, these findings indicate that ATL-III attenuates selected α-SMA-associated features of TGF-β1-induced myofibroblast differentiation. The representative immunofluorescence images were interpreted qualitatively, whereas the significant reduction in α-SMA protein expression at 100 µM was supported by Western blot densitometry. 

### 2.4. Effects of ATL-III on TGF-β1-Induced Intracellular Signaling in Human Corneal Keratocytes

**Figure 4:**
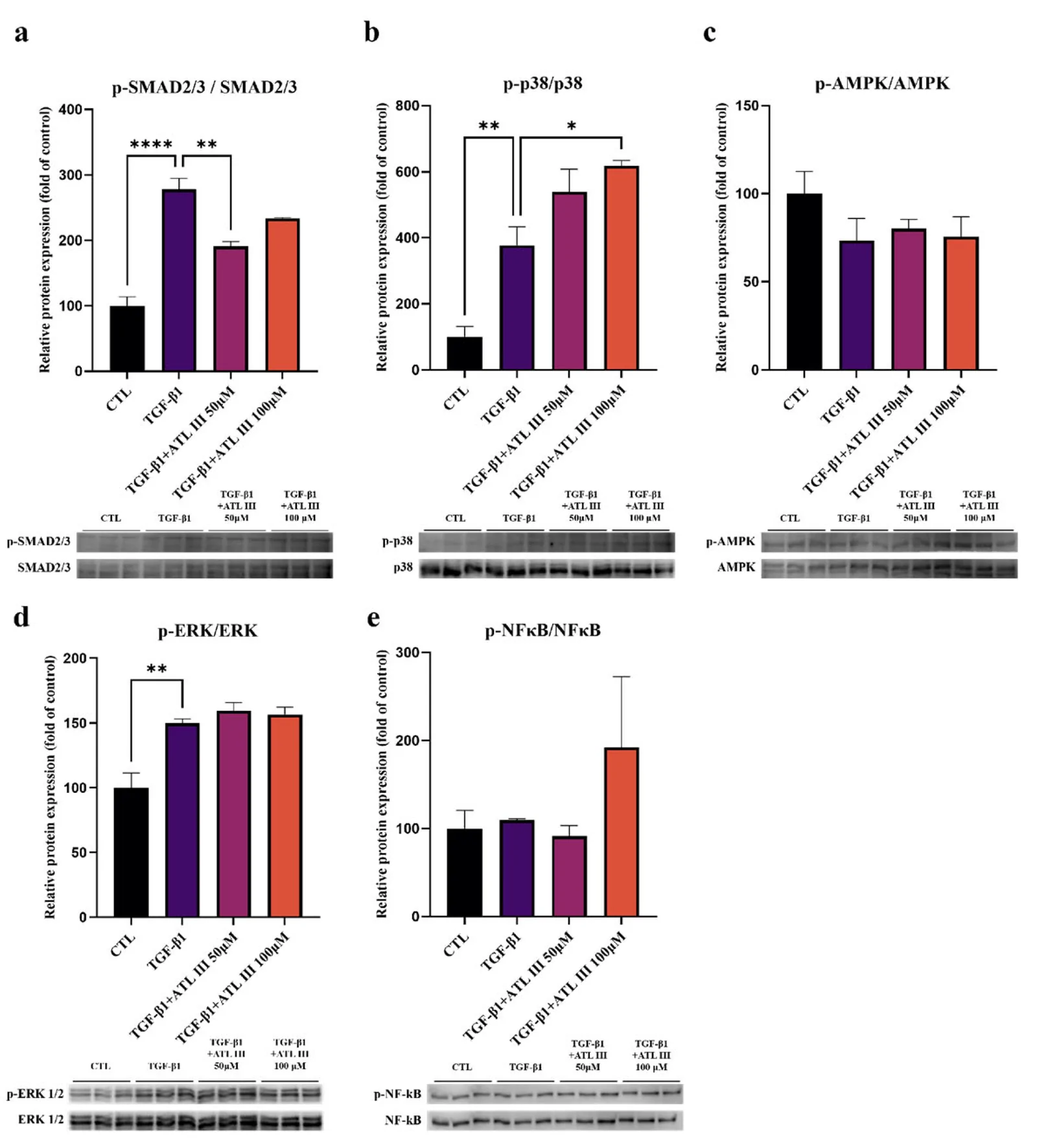
**Effects of ATL-III on TGF-β1-induced intracellular signaling in primary human corneal keratocytes.** Primary human corneal keratocytes were treated with TGF-β1 (10 ng/mL) alone or in combination with ATL-III (50 or 100 µM) for 1 h. Representative Western blot images and corresponding densitometric analyses are shown for (**a**) phosphorylated SMAD2/3 (p-SMAD2/3) and total SMAD2/3, (**b**) phosphorylated p38 MAPK (p-p38 MAPK) and total p38 MAPK, (**c**) phosphorylated AMPK (p-AMPK) and total AMPK, (**d**) phosphorylated ERK1/2 (p-ERK1/2) and total ERK1/2, and (**e**) phosphorylated NF-κB (p-NF-κB) and total NF-κB. Phosphorylated protein levels were normalized to their corresponding total protein levels and expressed relative to the untreated control group. n = 3 independent experiments. Statistical significance was determined by one-way ANOVA followed by Dunnett’s multiple-comparison test, with the TGF-β1-treated group used as the reference group for treatment comparisons. * *p* < 0.05, ** *p* < 0.01, and **** *p* < 0.0001.

To characterize early signaling responses associated with ATL-III treatment, phosphorylation of key TGF-β1-related signaling proteins was evaluated 1 h after treatment. TGF-β1 increased SMAD2/3 phosphorylation, which was significantly reduced by 50 µM ATL-III; however, this reduction was not statistically significant at 100 µM. In contrast, p38 MAPK phosphorylation was further increased at 100 µM ATL-III. No significant ATL-III-mediated changes in AMPK, ERK1/2, or NF-κB phosphorylation were detected relative to TGF-β1 alone at the 1-h time point (Figure 4).The numerical data supporting Figure 1, Figure 2, Figure 3 and Figure 4 are included in the Supplementary Materials. 

Taken together, these findings demonstrate distinct and non-uniform concentration-dependent signaling responses to ATL-III. The phosphorylation data alone do not establish the functional involvement of any individual pathway in the downstream phenotypic changes.

## 3. Discussion

Myofibroblast differentiation is an important component of corneal stromal wound healing, whereas persistent myofibroblast activity contributes to pathological stromal remodeling and loss of transparency [5,6,8]. In the present study, ATL-III reduced TGF-β1-induced ACTA2 expression and α-SMA protein expression in primary cultured human corneal stromal cells. COL1A1 expression was also significantly reduced at 100 µM, whereas CTGF and FN1 showed nonsignificant decreasing trends. These findings support an effect of ATL-III on selected features of TGF-β1-induced myofibroblast differentiation but do not establish generalized suppression of a complete corneal fibrotic phenotype [20]. Similar suppression of TGF-β-induced corneal fibroblast-to-myofibroblast differentiation has been reported with other bioactive compounds, including andrographolide [20].

Recent studies have emphasized the complexity of TGF-β-driven corneal stromal cell differentiation and the involvement of interconnected canonical and non-canonical signaling networks [7,8,21]. Consistent with these observations, the signaling responses in the present study were not uniformly concentration-dependent.

Importantly, the attenuation of myofibroblast-associated phenotypes was accompanied by distinct early signaling changes rather than uniform suppression of a single pathway, suggesting a complex and concentration-dependent cellular response to ATL-III.

TGF-β1 stimulation produced a pronounced early increase in SMAD2/3 phosphorylation, consistent with the established role of canonical TGF-β/SMAD signaling in corneal myofibroblast differentiation [7,8,22,23,24,25,26]. Similar pharmacological modulation of TGF-β/SMAD signaling has also been reported in human conjunctival fibroblasts, where inhibition of SMAD2 phosphorylation was associated with reduced myofibroblast transdifferentiation and fibrosis-associated markers [27].

ATL-III significantly reduced SMAD2/3 phosphorylation at 50 µM. However, this response was not observed at 100 µM, whereas the reduction in α-SMA protein expression was more pronounced at 100 µM. Therefore, the present findings demonstrate an association between ATL-III treatment and altered SMAD2/3 phosphorylation but do not establish inhibition of SMAD2/3 signaling as the mechanism responsible for the phenotypic changes. Pathway-specific pharmacological or genetic studies will be required to establish causality.

No significant ATL-III-mediated change in AMPK phosphorylation was detected at the 1-h signaling time point. Because AMPK activity is temporally regulated, the absence of a significant change at this single early time point does not exclude a potential contribution of AMPK at later stages. Accordingly, the present data are insufficient to determine the functional involvement of AMPK in the cellular response to ATL-III.

TGF-β1 increased ERK1/2 phosphorylation relative to control. ATL-III did not significantly reduce ERK1/2 phosphorylation relative to TGF-β1 alone at the 1-h time point. Therefore, the present data do not provide evidence for ERK1/2 suppression under these experimental conditions, although involvement of this pathway cannot be excluded.

Unexpectedly, p38 MAPK phosphorylation was further increased by 100 µM ATL-III. Previous studies have implicated p38 MAPK in TGF-β-induced corneal myofibroblast differentiation [28]. This response occurred despite reductions in ACTA2, COL1A1, and α-SMA observed under the 48-h phenotypic conditions. Because phosphorylation was assessed only at an early 1-h time point and no pathway-specific intervention was performed, the functional significance of the increased p38 phosphorylation cannot be determined from the present study. Further concentration- and time-dependent analyses are required to clarify this response. The dissociation between early p38 phosphorylation and the later reduction in myofibroblast-associated phenotypes further indicates that a single early phosphorylation event may not directly predict the downstream phenotypic response.

No significant ATL-III-mediated alteration in NF-κB phosphorylation was detected at the 1-h time point. This finding should not be interpreted as excluding NF-κB involvement at other time points or under different experimental conditions.

The cytotoxicity analysis demonstrated that ATL-III did not significantly affect keratocyte viability within the concentration range used in this study. In addition, cell viability remained unchanged after 48 h of treatment under the same exposure conditions used for the phenotypic analyses. These findings support the interpretation that the observed reductions in α-SMA expression and other fibrosis-associated markers were unlikely to result from nonspecific cytotoxicity or loss of cell viability.

These observations are limited to the ATL-III concentrations, exposure durations, and in vitro model examined in this study and should not be extrapolated to other doses, longer exposure periods, or in vivo conditions.

The treatment durations were selected according to the biological endpoint being evaluated. The 1-h treatment was used exclusively to evaluate early phosphorylation responses following TGF-β1 stimulation and was not intended to induce or assess myofibroblast differentiation. In contrast, transcriptional and phenotypic endpoints, including fibrosis-associated gene expression and α-SMA expression, were assessed after 48 h of TGF-β1 exposure. The initial 24-h viability assay served as a preliminary concentration-screening step, while an additional 48-h viability assessment was performed to confirm cellular tolerance over the same exposure duration used for the phenotypic experiments.

Several limitations should be acknowledged. First, the present study was conducted exclusively in primary cultured human corneal stromal cells in vitro, and the observed effects remain to be validated in vivo. Although the cells were derived directly from human donor corneal stromal explants and maintained under serum-free culture conditions, their phenotype was not independently characterized using keratocyte-specific markers at passages 3–5. Therefore, culture-associated phenotypic drift toward a fibroblast-like state cannot be excluded.

Second, extracellular matrix remodeling was evaluated primarily at the transcriptional level. Although COL1A1, CTGF, and FN1 were assessed by qPCR, broader protein-level validation, including collagen I, collagen III, CTGF, and EDA-fibronectin, was not performed. The present findings should therefore be interpreted as changes in selected myofibroblast- and fibrosis-associated features rather than comprehensive suppression of a fibrotic phenotype.

Third, signaling responses were evaluated at a single early 1-h time point. The reduction in SMAD2/3 phosphorylation at 50 µM was not reproduced at 100 µM and did not parallel the stronger reduction in α-SMA protein observed at 100 µM, while p38 MAPK phosphorylation was further increased at 100 µM. Because pathway-specific inhibition or genetic manipulation was not performed, causal involvement of SMAD2/3, p38 MAPK, AMPK, ERK1/2, or NF-κB cannot be inferred from phosphorylation changes alone.

Fourth, the initial cell-viability experiment was used to identify tolerated concentrations rather than to establish a minimum effective dose. Therefore, a complete concentration–response relationship, including phenotypic evaluation at lower ATL-III concentrations such as 10 µM, remains to be established.

Finally, the absence of detectable cytotoxicity is limited to the concentrations, exposure durations, and in vitro model examined here and should not be extrapolated to other doses, longer exposure periods, or in vivo conditions.

## 4. Materials and Methods

### 4.1. Primary Human Corneal Keratocyte Culture

Corneal keratocytes were isolated from human donor corneal stromal tissue under the protocol determined to be exempt from Institutional Review Board review by the Samsung Medical Center Institutional Review Board (IRB No. 2025-05-064). All procedures involving human tissue were conducted in accordance with the Declaration of Helsinki. Briefly, the corneal stroma was dissected and minced into small tissue fragments under sterile conditions. The minced stromal tissue fragments were placed onto FNC-coated culture plates and maintained as explant cultures to allow keratocytes to migrate out from the tissue and expand on the culture plate surface.

The cells were cultured in Dulbecco’s modified Eagle’s medium/Ham’s F-12 (DMEM/F12; [Gibco, 11320033]) supplemented with 10 ng/mL insulin-like growth factor, 0.5 mM L-ascorbic acid, 1% insulin–transferrin–selenium, and 1% antibiotics (Gibco, 15240062). Cells were maintained at 37 °C in a humidified atmosphere containing 5% CO_2_, and the culture medium was replaced every 2–3 days. Cells between passages 3 and 5 were used for all experiments. ATL-III (Chemfaces, Wuhan, China; CFN99946; purity ≥ 98%) was dissolved in DMSO to prepare a 100 mM stock solution and stored at 4 °C until use. Working solutions were freshly prepared by dilution in culture medium, and the final concentration of DMSO was maintained at 0.05% in all relevant groups. Cells were derived from human corneal stromal explants and maintained under serum-free culture conditions; however, keratocyte-specific phenotypic markers were not independently assessed at these passages.

Unless otherwise specified, cells were supplement-starved for 24 h in DMEM/F12 without supplements before treatment. For fibrosis experiments, cells were assigned to four experimental groups: untreated control (CTL), TGF-β1 (10 ng/mL), TGF-β1 (10 ng/mL) plus ATL-III 50 µM (ATL-III 50 µM), and TGF-β1 (10 ng/mL) plus ATL-III 100 µM (ATL-III 100 µM). For signaling analyses, cells were harvested 1 h after treatment to evaluate early phosphorylation events. For transcriptional and myofibroblast-associated phenotypic analyses, cells were exposed to the indicated treatments for 48 h.

### 4.2. Cell Viability Assay

The cytotoxicity of ATL-III was evaluated using a D-Plus Cell Counting Kit (CCK.3000; Dongin LS, Seoul, Republic of Korea). Primary human corneal keratocytes were seeded in 96-well plates and cultured until they reached approximately 90% confluence.

To determine the concentration range of ATL-III for subsequent experiments, cells were treated with increasing concentrations of ATL-III (0, 2, 5, 10, 25, 50, 100, and 120 µM) for 24 h. In a separate experiment, cell viability was assessed after 24 h and 48 h of treatment to evaluate cellular tolerance over the same exposure duration used for the phenotypic analyses.

Following treatment, D-Plus CCK reagent was added to each well according to the manufacturer’s instructions. Absorbance was measured at 450 nm using a SpectraMax microplate reader (Molecular Devices, San Jose, CA, USA), and cell viability was expressed as a percentage relative to the untreated control group.

### 4.3. Quantitative Real-Time PCR

Primary cultured human corneal keratocytes were supplement-starved for 24 h and subsequently treated with TGF-β1 (10 ng/mL) alone or in combination with ATL-III at 50 or 100 µM. The untreated group served as the control.

Total RNA was extracted using TRIzol reagent (Invitrogen/Thermo Fisher Scientific, Waltham, MA, USA; catalog no. 15596018) according to the manufacturer’s instructions. RNA concentration and purity were determined using a NanoDrop spectrophotometer (Thermo Fisher, ND-2000UV). Complementary DNA was synthesized from 1 µg of total RNA using random primers for reverse transcription (Toyobo, ReverTra Ace qPCR RT Master Mix, Osaka, Japan, catalog no. FSQ-201).

Quantitative real-time PCR was performed using LUNA Universal qPCR mastermix (New England Biolabs, Ipswich, MA, USA, catalog no. M3003) on a LightCycler 480 (Roche) system. The reaction conditions were as follows: 95 °C/5 min; 45 cycles of 95 °C/10 s, 55 °C/10 s, 72 °C/10 s; and 72 °C/10 min. Melting-curve analysis was performed after amplification to confirm the specificity of the PCR products. Primer amplification efficiencies were confirmed to be within an acceptable range. Relative gene expression was calculated using the 2^−ΔΔCt method and normalized to GAPDH. The primers used in this study were synthesized by Macrogen (Seoul, Republic of Korea), and their sequences are listed in Table 1.

### 4.4. Immunofluorescence Staining

To evaluate myofibroblast differentiation and cytoskeletal remodeling, primary cultured human corneal keratocytes were cultured on coverslips and subjected to 24 h of supplement starvation before treatment. Cells were subsequently treated with TGF-β1 (10 ng/mL) alone or in combination with ATL-III (50 or 100 µM) for 48 h.

Following treatment, cells were washed with phosphate-buffered saline (PBS) and fixed with 4% PFA for 24 h at 4 °C. This fixation duration reflects the actual protocol used in this study, and all experimental groups were fixed and processed under identical conditions. After washing with PBS, cells were permeabilized with 0.1% Triton X-100 in PBS and blocked with 2% bovine serum albumin (BSA) for 30 min at room temperature. Cells were incubated with a primary antibody against α-SMA overnight at 4 °C. After washing with PBS, cells were incubated with the appropriate fluorescence-conjugated secondary antibody for 1 h at room temperature in the dark. F-actin was visualized using phalloidin, and nuclei were counterstained with DAPI-containing mounting medium. Fluorescence images were acquired using a Leica DMi8 microscope (Leica Microsystems, Wetzlar, Germany). Identical acquisition settings were used across all experimental groups.

### 4.5. Western Blot Analysis

Primary cultured human corneal keratocytes were subjected to 24 h of supplement starvation before treatment with TGF-β1 (10 ng/mL) alone or in combination with ATL-III (50 or 100 µM). For analysis of early intracellular signaling, cells were harvested 1 h after treatment. For analysis of myofibroblast differentiation, cells were harvested 48 h after treatment. Cells were washed with ice-cold PBS and lysed in RIPA buffer supplemented with protease and phosphatase inhibitors. Cell lysates were incubated on ice for 30 min and subsequently centrifuged at 14,000 × g for 10 min at 4 °C. The supernatants were collected, and protein concentrations were determined using the Bradford protein assay. Equal amounts of protein (20 µg per lane) were mixed with 4× sample buffer, adjusted to an equal final loading volume, and denatured at 100 °C for 5 min. Proteins were separated by SDS-PAGE and transferred onto PVDF membranes.

Following transfer, membranes were blocked with 5% BSA for 30 min and incubated overnight at 4 °C with primary antibodies against the following proteins: phospho-SMAD2/3 (Cellsignaling, catalog no. 8828), SMAD2/3 (Cellsignaling, catalog no. 3102), phospho-AMPK (Cellsignaling, catalog no. 2531), AMPK (Cellsignaling, catalog no. 2532), phospho-NF-κB (Cellsignaling, catalog no. 3033), NF-κB (Cellsignaling, catalog no. 8242), phospho-p38 MAPK (Cellsignaling, catalog no. 9211), p38 MAPK (Cellsignaling, catalog no. 9212), phospho-ERK1/2 (Cellsignaling, catalog no. 9101), ERK1/2 (Cellsignaling, catalog no. 9102), α-SMA (Cellsignaling, catalog no. 14968), GAPDH (Cellsignaling, catalog no. 5174). After washing with Tris-buffered saline containing Tween-20 (TBST), membranes were incubated with the appropriate horseradish peroxidase-conjugated secondary antibodies for 1 h at room temperature. Protein bands were visualized using an enhanced chemiluminescence substrate and captured using the ChemiDoc imaging system (BioRad Laboratories, Hercules, CA, USA).

Band intensities were quantified using ImageJ (version 1.54, National Institutes of Health, Bethesda, MD, USA). For phosphorylation-dependent signaling analyses, each phosphorylated protein was normalized to its corresponding total protein (p-SMAD2/3/SMAD2/3, p-p38/p38, p-AMPK/AMPK, p-ERK1/2/ERK1/2, and p-NF-κB/NF-κB). This approach was used to assess relative phosphorylation while accounting for total target-protein abundance. α-SMA, which was evaluated as total protein expression rather than a phosphorylation ratio, was normalized to GAPDH.

### 4.6. Statistical Analysis

Data are presented as mean ± SEM. Statistical analyses were performed using GraphPad Prism (version 8.0, GraphPad Software, Boston, MA, USA). Comparisons among multiple experimental groups were performed using one-way analysis of variance (ANOVA) followed by Tukey’s or Dunnett’s multiple-comparison test, as appropriate.

## 5. Conclusions

In conclusion, this study provides preliminary evidence that ATL-III attenuates selected features of TGF-β1-induced myofibroblast differentiation in primary human corneal stromal cells under the experimental conditions examined. ATL-III significantly reduced ACTA2 expression at 50 and 100 µM, COL1A1 expression at 100 µM, and α-SMA protein expression at 100 µM, whereas CTGF and FN1 showed nonsignificant decreasing trends. The phenotypic attenuation was accompanied by distinct early signaling responses, including reduced SMAD2/3 phosphorylation at 50 µM and increased p38 MAPK phosphorylation at 100 µM. These non-uniform responses suggest a complex, concentration-dependent signaling profile rather than uniform suppression of a single pathway and do not establish a causal mechanism for the observed phenotypic changes. Additional protein-level validation, pathway-specific experiments, concentration- and time-dependent analyses, and in vivo studies are required to determine whether these preliminary effects translate into modulation of pathological corneal stromal remodeling.

## Figures and Tables

**Table 1:** Primer sequences used for quantitative real-time PCR.

Gene	Forward	Reverse
ACTA2	ctatgagggctatgccttgc	ttctccttgatgtcccggac
COL1A1	cggaggagagtcaggaag	acacaaggaacagaacagtc
CTGF	ctcctgcaggctagagaacg	gatgcactttttgcccttctt
Fibronectin	ctggccgaaaatacattgtaa	ccacagtcgggtcaggag

## Data Availability

The numerical data supporting the findings of this study are provided in the Supplementary Materials. Further inquiries can be directed to the corresponding author.

## Supplementary Materials

The following supporting information can be downloaded at: https://www.mdpi.com/article/10.3390/molecules31214040/s1. Minimal Dataset: numerical data supporting Figure 1, Figure 2, Figure 3 and Figure 4.
